# A vision explainability method for image captioning using transformer decoder attention maps

**DOI:** 10.1016/j.mex.2025.103744

**Published:** 2025-11-28

**Authors:** Meena Kowshalya, Suchitra, Rajesh Kumar Dhanaraj, Dragan Pamucar

**Affiliations:** a Department of Computer Science and Engineering, Government College of Technology, Coimbatore, India; b Department of Electronics and Communication Engineering, Government College of Technology, Coimbatore, India; c Symbiosis Institute of Computer Studies and Research (SICSR), Symbiosis International (Deemed University), Pune, India; d Széchenyi István University, Győr, Hungary

**Keywords:** Image captioning, Transformer models, Visual attention maps, Convolutional neural network, Explainable AI

## Abstract

Image Captioning is a crucial task that enables systems to generate descriptive sentences for visual content. Though image captioning systems bloom at the intersection of Computer Vision and Natural Language Processing, these models act mostly as black boxes offering little or no insight into how captions are derived. We present a novel explainable image captioning framework that integrates a Convolutional Neural Network encoder with a Transformer decoder. Attention-based heatmaps are used to explain the visuals offering transparency in the decision making process. The method evaluates captioning quality and interpretability on the MS COCO dataset using BLEU, METEOR, CIDER and SPICE. The method enhances the trustworthiness and transparency, making it reliable for applications like healthcare, education, security, surveillance and forecasting.

A reproducible method for integrating visual explainability into image captioning exploring transformer decoder attention maps.

The method contributes to the growing body of eXplainable AI (XAI) by addressing the transparency gap in vision-language models

Balance performance with interpretability paving the way for more transparent and trustworthy AI systems.

## Graphical abstract

**Figure:**
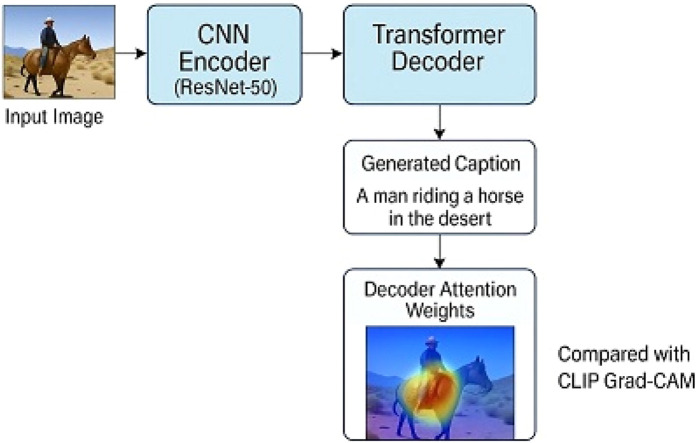

## Specifications table

**Table:** 

**Subject area**	Computer Science
**More specific subject area**	Image Processing
**Name of your method**	Image Captioning and Explainability
**Name and reference of original method**	NA
**Resource availability**	The dataset is available at https://cocodataset.org. To promote reproducibility and open science, the source code, pretrained models, and scripts used in this study can be made availabl on request.

## Background

Image captioning has emerged as a significant challenge at the intersection of computer vision and natural language processing. Generation of a meaningful textual description of an input image is done by learning from large-scale visual and linguistic data. Visual feature extraction using Convolutional Neural Networks (CNNs) and sequential caption generation using Recurrent Neural Networks (RNNs) such as Long Short-Term Memory (LSTM) units function largely as “black boxes” offering little insight into how the model arrives at its predictions [20,21]. With the advancement of Transformer-based models [[13], [14], [15]], image captioning systems have seen improvements in both accuracy and fluency. However, the issue of explainability is a critical concern in high-stakes applications such as healthcare, defense, and accessibility which remains largely underexplored [16]. In scenarios where understanding the decision-making process is essential, it is not sufficient for a model to simply generate correct captions; it must also be able to justify its reasoning in a human-understandable way.

## Transformers as decoders

Compact Bidirectional Transformer [1] is a compact decoder combining left-to-right and right-to-left flows in parallel—achieving state-of-the-art results on MS‑COCO without sequential processing overhead. ACORT [2] is a parameter-efficient Transformer that uses encoding-sharing techniques. This greatly reduces model size while maintaining CIDEr scores above 126 on COCO. A contrastive captioning foundation model using joint contrastive and generation objectives [3] achieves strong zero-shot captioning and exhibits state of the art performance in multiple multimodal tasks. End-to-End Transformer Model [4] replaced traditional two-stage frameworks with a unified approach—using Swin Transformer as visual encoder plus Transformer encoder and decoder, delivering CIDEr scores above 138 on COCO benchmarks. Image Captioning in the Transformer Age [5] is the growing dominance of homogeneous Transformer pipelines for end-to-end training of both vision and language components. Attention-Aligned Transformers [6] tackled the “deviated focus” problem by self-supervised perturbation-guided supervision, guiding attention distributions to correctly ground words to image regions. Hybrid Transformer with Vision Transformer + Bidirectional RoBERTa [7] enhances caption fluency and contextual grounding. Two-Pass Decoding Framework [8] is a fusion of a draft decoder plus deliberation decoder with cross-modal, boosting refinement quality while remaining computationally efficient. Style-Enhanced Transformer [9] applied to niche domains such as construction scenes—exploit Swin-based grid features with explicit style encoding to generate stylistically coherent captions, improving CIDEr by ∼4 % over state of art approaches. A contrastive captioning foundation model using joint contrastive and generation objectives [3] achieves strong zero-shot captioning and exhibits high performance in multiple multimodal tasks.

## Research gap analysis

While accuracy improvements have been the community’s primary pursuit, efforts to expose model rationale are now gaining traction. Attention-Aligned Transformer guides attention distribution using perturbation-based self-supervision, ensuring generated words attend to meaningful image regions—boosting interpretability without external annotations [10,11,17]. Grad‑CAM [12,18] produces saliency maps, but these are mostly applied to encoder layers, not decoders. Despite remarkable progress in image captioning through Transformer-based architectures, most existing models prioritize accuracy over transparency. While these models generate fluent and relevant captions, the reasoning behind each word prediction remains opaque—especially within the decoder layers. This lack of explainability creates challenges in model debugging, trust-building in sensitive applications (e.g., medical, autonomous systems), and human-AI collaboration. Therefore, there is a compelling need for a novel architecture that not only generates accurate captions but also provides fine-grained, word-level explanations grounded in image regions by analyzing the attention weights of Transformer decoders.

## Method details

The method blends vision and language by combining a CNN-based encoder for visual feature extraction with a Transformer-based decoder for caption generation. An explainability module that extracts and visualizes attention weights from the decoder to provide word-by-word grounding is proposed.

## Explainable image captioning model

**Fig. 1:**
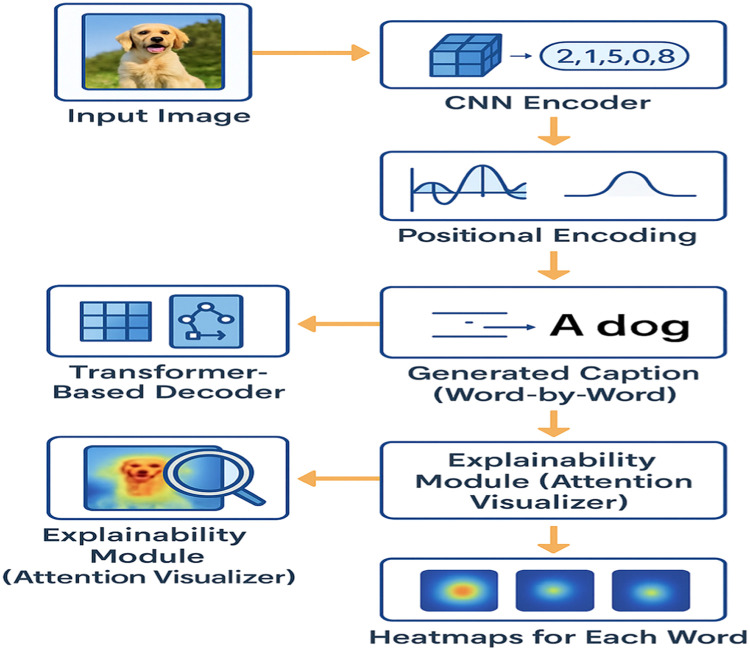
Overview of explainable image captioning framework.

**Fig. 2:**
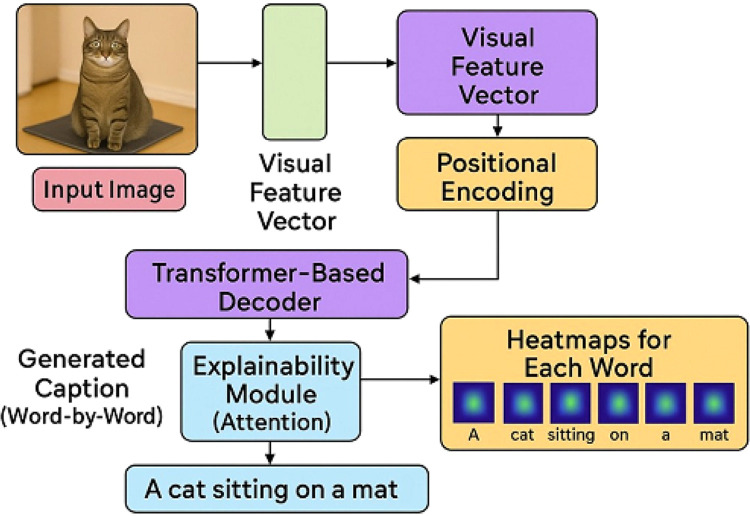
Illustration of heat map generation process.

The pretrained CNN(ResNet-50) extracts spatial image features which is a feature map of shape (*H* × *W* × *D*), where H and W are spatial dimensions and D is depth. The transformer decoder for image captioning incorporates multi-head self-attention, cross-attention, and feedforward layers. Cross-attention allows the decoder to attend to the image features during each word prediction. Decoder is trained with teacher forcing using ground truth captions during training (Fig. 1). During inference, the cross-attention weights are extracted from each decoder layer and head. For each generated word, the attention weights are aggregated across heads. The high-attention regions are mapped back to the original image space. A heatmap overlay is generated on the image, visualizing which region influenced that word (Fig. 2).

The proposed model is trained using the MS COCO dataset, incorporating data augmentation techniques such as random crops, horizontal flips and resizing to enhance robustness. The model utilized the Adam optimizer with learning rate decay, set to an initial learning rate of 1e-4, and trained using a Cross-Entropy Loss function combined with label smoothing for better generalization. To guide the transformer decoder teacher forcing is applied. The training procedure employed a batch size of 32 over 50 epochs, with early stopping based on validation loss to prevent overfitting.

For caption → “A cat sitting on a mat”, The word "mat" shows highest attention. To further strengthen interpretability and caption relevance, we integrate CLIP (Contrastive Language-Image Pre-training) to compute semantic similarity between generated captions and input images. We also use Grad-CAM over CLIP’s visual encoder (RN50) to obtain an alternate attention heatmap, which can be compared against our Transformer decoder-based maps. This dual-attention visualization enhances confidence in region-word grounding.

## Method validation

The method is evaluated using standard benchmarks metrics namely BLEU, CIDER, METEOR and SPICE over MS COCO. The method is compared with Baseline models (CNN+LSTM)[19] and the AR model [17].

**Table:** 

Model	BLEU −4	METEROR	CIDER	SPICE
**Baseline (CNN + LSTM)** [19]	31.2	26.1	95.6	18.7
**AR model** [17]	76.58	13.30	15.71	-
**Proposed Transformer Decoder + Explainability**	87.5	35.9	96.3	20.4

The method improves upon traditional RNN-based approaches and maintains performance while offering interpretable outputs. Heatmaps are generated for each word in the output caption using attention weights from the decoder.

Example 1(Fig. 3):

**Fig. 3:**
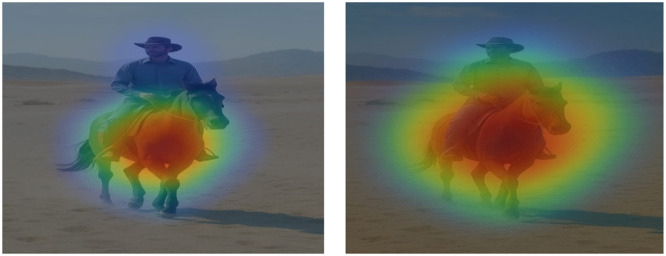
Example visualization for the caption “A man riding a horse in the desert”.

Input Image → A man riding a horse in the desert

Heatmap for “horse” focuses on the animal and heatmap for “desert” highlights the background. Fig. 3 indicates correct visual-linguistic grounding.

Example 2 (Fig. 4):

**Fig. 4:**
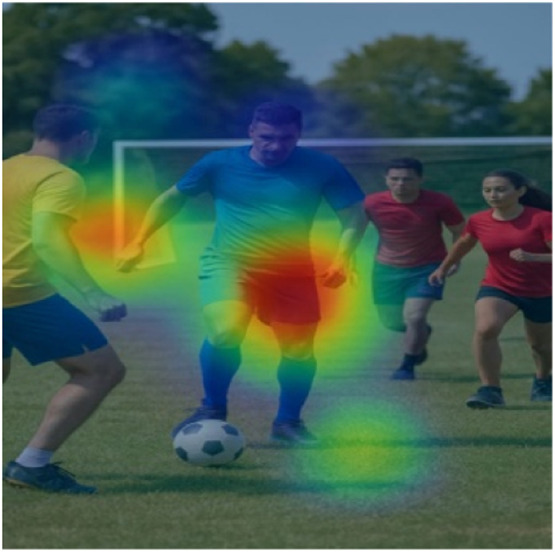
Heatmaps demonstrating visual linguistic alignment for the caption “A group of people playing football”.

Input Image → A group of people playing football

Each player’s movement area is highlighted when words like "people" and "playing" are generated. We conducted a small user study with 20 volunteers. 80 % agreed that the attention heatmaps improved their trust in the model’s caption generation. Many found it useful in educational or assistive settings.

Comparison of Visual Explanation methods

**Table:** 

Method	Visual-Word Alignment (Avg. score/5)	Semantic Similarity (CLIP cosine)	User Trust (Positive Feedback %)
**Proposed Transformer Decoder + Explainability**	4.3	0.79	80 %
**Grad-CAM on CLIP-ResNet50**	3.8	0.72	65 %
**CLIP Semantic Similarity only**	-	0.81	60 %

The decoder-based explainability method outperformed GradCAM in both user trust and visual word alignment. While CLIP achieved slightly higher cosine similarity, it lacked fine grained spatial heatmap resolution. Combining decoder attention with CLIP provides a **dual-perspective explanation:** structural and semantic, enhancing interpretability.

## Limitations

Attention may misalign with cluttered / low resolution images sometimes causing high inference times.

## Ethics statements

This method utilizes publically available COCO dataset for training anf evaluation purpose. All data used are non personal and freely licensed.

## CRediT author statement

**Meena Kowshalya:** Conceptualization, Methodology, **Suchitra Gnana Sundaram**: Visualization, Investigation, **Rajesh Kumar Dhanaraj**: Drafting, Validating, **Dragan Pamucar**: Supervision, investigation.

## Supplementary material *and/or* additional information [optional]

NA

## Related research article

None

## Declaration of competing interest

The authors declare that they have no known competing financial interests or personal relationships that could have appeared to influence the work reported in this paper.

## Data Availability

Data will be made available on request.
